# *CHEK2 *1100delC in patients with metachronous cancers of the breast and the colorectum

**DOI:** 10.1186/1471-2407-6-64

**Published:** 2006-03-15

**Authors:** Anna Isinger, Misha Bhat, Ake Borg, Mef Nilbert

**Affiliations:** 1 Department of Oncology, Institute of Clinical Sciences, Lund University, 221 85 Lund, Sweden

## Abstract

**Background:**

Development of multiple primary tumors is a hallmark of hereditary cancer. At least 1/10 of breast cancers and colorectal cancers occur because of heredity and recently the cell cycle kinase 2, *CHEK2 *1100delC allele has been identified at a particularly high frequency in families with hereditary breast and colorectal cancer.

**Methods:**

We utilized the Southern Sweden population-based cancer registry to identify women with double primary breast and colorectal cancer and sequenced tumor material in order to assess the contribution of the *CHEK2 *1100delC to the development of such metachronous tumors.

**Results:**

Among the 75 patients successfully analyzed, 2 (2.5%) carried the *CHEK2 *1100delC allele. which was not significantly different (p = 0.26) from the 1% (3/300) carriers identified in the control group.

**Conclusion:**

In summary, our data suggest that the *CHEK2 *1100delC is not a major cause of double primary breast and colorectal cancer in Sweden, which suggests that this patient group should not routinely be screened for the *CHEK2 *1100delC variant.

## Background

At least 10% of both breast cancers and colorectal cancers are estimated to develop because of heredity. The *BRCA1 *and *BRCA2 *genes are the major causes of hereditary breast and ovarian cancer, but the underlying genetic defect remains unresolved in the majority of the families with familial or hereditary breast cancer [1,2]. In colorectal cancer, familial adenomatous polyposis due to mutations in *APC *and the hereditary nonpolyposis colorectal cancer (HNPCC) syndrome caused by mutations in the mismatch-repair genes *MLH1, MSH2, MSH6 *and *PMS2 *are the underlying causes of many families with hereditary colorectal, whereas the genetic defects remains unknown in most of the families with hereditary colorectal cancer with higher age at onset and lack of other associated tumor types [3,4]. Searches for additional high-penetrant disease-causing genes in these cancer types have so far not been successful and it is likely that low-penetrant genetic variants may contribute a substantial fraction of hereditary breast cancer and colorectal cancer [3,5].

Double-strand DNA breaks lead to activation of the cell cycle checkpoint kinase 2, CHEK2 [GenBank:AL121825], through ATM phosphorylation [6]. CHEK2 plays an important role in cell cycle regulation and DNA damage repair, processes that are central in prevention of tumor development. The *CHEK2 *1100delC mutation leads to premature termination and abrogates kinase activity. This variant allele occurs in about 1% of normal populations, but has been identified at increased frequencies (1.2–1.9%) in individuals with breast cancer [7,8]. An approximately 2-fold increased risk of breast cancer has been associated with the *CHEK2 *1100delC mutation [8]. Higher frequencies, 5.1–11.4%, of the *CHEK2 *1100delC allele have by some investigators been reported in non-*BRCA1/2 *families with hereditary breast cancer [8-11], although other studies from e.g. Australia and Spain have not found an increased frequency in such families [12,13]. These differences may be ascribed to geographical variations as well as to different sample sets, particularly regarding the number of family members affected, age at onset, and selection of e.g. families with male breast cancer. Since the *CHEK2 *1100delC variant does not show clear co-segregation with disease it is considered to be a modifier, and may act in cooperation with yet unidentified high-penetrance genes or together with multiple low-penetrant genes as part of polygenic inheritance.

In studies from Finland and the Netherlands the *CHEK2 *1100delC has been reported at frequencies of 1.6–2.6% among individuals with colorectal cancer, which is not significantly higher than in unaffected individuals from the population, but these studies may be compatible with a risk of 1.5–2.0 for development of colorectal cancer among carriers [14-16]. A particularly high frequency of the *CHEK2 *1100delC allele has been reported in families with hereditary breast and colorectal cancer where 10/55 Dutch families (18%) carried the mutation [16].

Since development of multiple primary tumors in an individual is a sign of hereditary cancer we utilized a population-based cancer registry to identify women who had developed breast cancer and colorectal cancer in order to assess the contribution of the *CHEK2 *1100delC mutation to the development of metachronous cancers of these types.

## Methods

### Patient material

Ethical approval was obtained from the ethics committee at the Lund University. We utilized the population-based cancer registry of the Southern swedish health care region (which currently has approximately 1.7 million inhabitants). The registry was established in 1958 and because of mandatory cancer registration for clinicians as well as for pathologists the registry is estimated to contain 98% of all cancers diagnosed in this area. All females diagnosed with at least one breast cancer and one colorectal cancer during the time period 1958–2000 were identified. Totally 96 patients were identified in whom breast cancer was the first tumor in 64 and colorectal cancer was the first tumor in 25, whereas the remaining 7 patients developed synchronous breast and colorectal cancer. The mean age at diagnosis of the first of tumor was 68 (range 40–95) years, breast cancer developed at mean age 70 (range 40–95) and colorectal cancer at age 74 (range 47–98). After omission of patients who were misclassified or from whom the tumor blocks could not be retrieved, 84 patients remained for analysis.

Tumor-containing paraffin-embedded tissue and normal tissue (e.g. resection borders or benign lymph nodes) were retrieved. Fresh sections were obtained and stained with Hematoxylin & Erythrosin in order to verify that tumor tissue was present in the samples. DNA was extracted from 3 × 10 μm paraffin sections using treatment with Proteinase K in 65°C for at least 2 hours and boiled for 10 minutes for enzyme inactivation, whereafter the samples were centrifuged and the aqueous phase was removed for use.

### dHPLC and sequencing

Mutation analysis was carried out using dHPLC (Transgenomic WAVE Nucleic Acid Fragment Analyzer System Model 3500HT) and samples with aberrant patterns were further analyzed by direct sequencing (Terminator Cycle Sequencing Reaction Kits version 3.1, ABI Prism 3100 Genetic Analyzer; Applied Biosystems). Due to homologous sequences primer design is complex for *CHEK2 *exon 10. The primers used were 5'-TGGCAAGTTCAACATTATTCCC-3' (forward) [17] and 5'-ATCACCTCCTACCAGTCTGTGC-3' (reverse) [10]. PCR amplification was performed in a final volume of 25 μl containing: 2.5 μl 10 × PCR buffer, 2–3 μl MgCl (25 mM), 0.5 μl dNTP (20 mM), 0.7 μl primer (10 μM) and 0.1 μl polymerase. PCR conditions are available from the authors upon request. Heteroduplex formation was performed by mixing tumor DNA with wild-type DNA, heating to 94°C and lowering the temperature by 1°C per minute until 45°C was reached.

## Results

**Figure 1:**
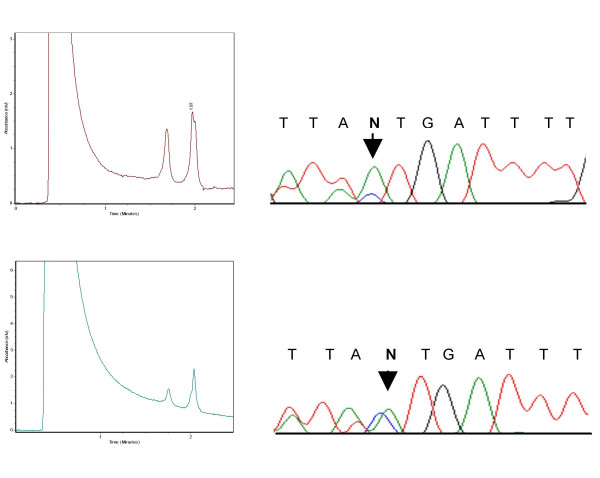
dHPLC and reverse sequence results that demonstrate the *CHEK2 *1100delC variant in the two patients. Both patients were heterozygous mutation carriers. The heteroduplex formations are seen as extra peaks in the dHPLC chromatogram.

Of the 84 patients, 75 (89%) were successfully analyzed. In total, 36 breast cancers and 67 colorectal cancers were analyzed and from 34 patients normal tissue was also analyzed. The majority of the patients developed breast cancer as their first tumor (table 1) and 68 of the patients developed other malignant tumors, most commonly malignant melanoma (n = 18), endometrial cancer (n = 12) and urinary bladder cancer (n = 6). The *CHEK2 *1100delC mutation was detected in 2 patients (2.5%) (table 2). Both the breast cancer and the colorectal cancer was analyzed and found to carry the mutation in these cases and, in case 17 the mutation was verified also in normal tissue. Chromatograms over the results are shown in figure 1.

In order to determine the expected frequency of the *CHEK2 *1100delC in the Southern swedish population dHPLC analysis and direct sequencing was performed from 300 healthy individuals with identification of the variant allele in 3/300 individuals, thus at a population frequency of 1% [18].

## Discussion

A subtype of familial breast cancer that includes colorectal cancer was recognized by Lynch *et al*. already in 1972 [19] and recently the *CHEK2 *1100delC mutation was proposed to represent a low-penetrant breast cancer susceptibility allele [10,11]. This variant has been identified at a particularly high frequency (18%) in families with a hereditary breast- and colorectal cancer phenotype [16]. Since development of multiple primary tumors is a hallmark of hereditary cancer and a high frequency of the *CHEK2 *1100delC had been described in hereditary breast and colorectal families, we assessed the contribution of this variant to the development of double primary breast and colorectal cancer in a population-based patient material. Among the 75 patients with metachronous tumors of the breast and the colorectum successfully studied, 2 (2.5%) carried the *CHEK2 *1100delC mutation compared to 1% in the control group [18]. These frequencies were not significantly different (p = 0.26), but the small size of the population-based patient material limits the strength of this comparison. However, the low frequency of this alteration in our material implies that development of metachronous breast cancer and colorectal cancer in women is *per se *not alone to identify individuals with a high likelihood of being carriers of the *CHEK2 *1100delC mutation. Although development of double primary tumors may be a sign of heredity it is not enough to recommend genetic analysis, though the family history of cancer should be carefully reviewed. Our findings are in line with previous studies that exclude *CHEK2 *1100delC as a major contributor to the breast and colorectal cancer phenotype [16]. Huang *et al*. [20] did not identify any *CHEK2 *1100delC mutation among 24 patients from the US with breast cancer and colorectal cancer. Also, in the study by Meijers-Heijboer *et al*. that primarily identified the link between *CHEK2 *1100delC and familial breast and colorectal cancer in Dutch families, the vast majority, 45/55 families, who fulfilled these criteria did indeed not carry this variant [16]. However geographical differences may influence the importance of the *CHEK *1100delC mutation.

Among the two women with the *CHEK2 *1100delC allele in our study, one presented with two separate, synchronous, breast cancers. An increased risk of multiple primary breast cancers (OR 5.7–6.5) has been reported in individuals carrying this *CHEK2 *1100delC allele [11,21,22]. However, the *CHEK2 *1100delC mutation has not only been suggested to act as a low-penetrant susceptibility gene in breast cancer families, but the *CHEK2 *I157T alteration has been proposed to act as a multiorgan cancer susceptibility allele based on observations of an increased risk for development of breast cancer, colon cancer, prostate cancer, thyroid cancer, and renal cancer in Polish families [23]. Both patients in our study, did in addition to breast cancer and colorectal cancer, develop a third tumor, two renal cancers at ages 49 and 73 (table 2). In the Swedish cancer registry 8–10% of the patients develop 2 or more malignancies (The Board of Social Health and Welfare, Cancer Incidence in Sweden 2003). The development of renal cancer in two of our patients was intriguing in relation to Cybulski *et al*. who identified an increased risk of renal cancer associated with *CHEK2*, albeit in carriers of the *CHEK2 *I157T variant [23]. However, since our study was registry-based no data on additional cancer cases in these families are available.

## Conclusion

In summary, our findings demonstrate that the *CHEK2 *1100delC occurs at a low frequency in Swedish women with double primary breast cancer and colorectal cancer, and thus suggests that development of these two tumor types is not sufficient to recommend mutation analysis of *CHEK2*.

## Conflict of interest statement

The author(s) declare that they have no competing interests.

## Authors' contributions

AI conceived of the study, performed the sequencing analysis, interpret data and drafted the manuscript. MB also carried out the sequencing. AB participated in its design and helped to draft the manuscript. MN conceived of the study, participated in its design and coordination and helped to draft the manuscript. All authors read and approved the manuscript.

## Pre-publication history

The pre-publication history for this paper can be accessed here:



## Figures and Tables

**Table 1:** Clinical data in the cohort analyzed (n = 75).

**Tumor type**	**Mean age (range) at diagnosis (years)**
Breast cancer	70 (40–94)
Colorectal cancer	74 (47–92)
Age at first cancer	68 (40–90)
Breast cancer as first cancer (n = 50)	68 (40–93)
Colorectal cancer as first cancer (n = 20)	70 (48–85)
Synchronous breast and colorectal cancer (n = 5)	70 (47–82)

**Table 2:** Clinical data from the two individuals carrying the *CHEK2 *1100delC mutation.

**Patient**	**Cancer type**	**Age (years)**
B17	Rectal cancer	69
	Renal cancer	73
	Breast cancer	74
B79	Synchronous breast cancer	47
	Colon cancer	48
	Renal cancer	49
